# W-box and G-box elements play important roles in early senescence of rice flag leaf

**DOI:** 10.1038/srep20881

**Published:** 2016-02-11

**Authors:** Li Liu, Wei Xu, Xuesong Hu, Haoju Liu, Yongjun Lin

**Affiliations:** 1 Key Laboratory for Economic Plants and Biotechnology, Kunming Institute of Botany, Chinese Academy of Sciences, Kunming, 650201, China; 2 Yunnan Key Laboratory for Wild Plant Resources, Kunming, 650201, China; 3 College of Life Sciences, Peking-Tsinghua Center for Life Sciences, Peking University, Beijing, 100871, China; 4 Department of Bioscience and Bioengineering, Jiangxi Agricultural University, Nanchang, 330045, China; 5 National Key Laboratory of Crop Genetic Improvement and National Centre of Plant Gene Research, Huazhong Agricultural University, Wuhan, 430070, China

## Abstract

Plant *cis*-elements play important roles in global regulation of gene expression. Based on microarray data from rice flag leaves during early senescence, we identified W-box and G-box *cis*-elements as positive regulators of senescence in the important rice variety Minghui 63. Both *cis-*elements were bound by leaf senescence-specific proteins *in vitro* and influenced senescence *in vivo*. Furthermore, combination of the two elements drove enhanced expression during leaf senescence, and copy numbers of the *cis*-elements significantly affected the levels of expression. The W-box is the cognate *cis-*element for WRKY proteins, while the G-box is the cognate *cis-*element for bZIP, bHLH and NAC proteins. Consistent with this, WRKY, bZIP, bHLH and NAC family members were overrepresented among transcription factor genes up-regulated according during senescence. Crosstalk between ABA, CTK, BR, auxin, GA and JA during senescence was uncovered by comparing expression patterns of senescence up-regulated transcription factors. Together, our results indicate that hormone-mediated signaling could converge on leaf senescence at the transcriptional level through W-box and G-box elements. Considering that there are very few documented early senescence-related *cis*-elements, our results significantly contribute to understanding the regulation of flag leaf senescence and provide prioritized targets for stay-green trait improvement.

Leaf senescence represents the final stage of leaf development and is a complicated process influenced by both external environmental factors and internal gene expression^1^. Extensive genome-wide studies have revealed thousands of senescence-associated genes as well as their responses to various inducers of senescence^2^,^3^,^4^. The most prominent data was obtained using high-resolution time-course microarray analysis during the development of a single Arabidopsis leaf over a 3-week period to senescence. The growth and biochemical changes during senescence were tracked, and transcription factors (TFs) that potentially function at critical time points to activate distinct pathways were identified^5^. A leaf senescence database (LSD, http://www.eplantsenescence.org/) was developed that contains 1145 senescence-associated genes (SAGs) from 21 species based on experimental evidence from the literature^6^. Hundreds of TFs, including members of WRKY, NAC, MYB, bHLH, and bZIP families, are found in the LSD, suggesting that leaf senescence is highly coordinated by complicated regulatory networks. To date, most of the progress in understanding senescence comes from the model dicot *Arabidopsis thaliana*, while limited information is known about rice SAGs.

In rice, few SAGs have been established to take part in the cellular activities underlying the senescence process. Among these, *NON-YELLOW COLORING 1* (*NYC1*), *NYC1-LIKE* (*NOL*) and *STAYGREEN* (*SGR*) activate chlorophyll degradation^7^,^8^,^9^. Rice *CORONATINE INSENSITIVE 1b* (*OsCOI1b*) encodes a homolog of the Arabidopsis JA receptor COI1. The *oscoi1b* T-DNA insertion mutant shows methyl jasmonate (MeJA) insensitivity as well as delayed senescence in both lab and field conditions^10^. In addition, overexpression of the SAG *OsWRKY42* in rice results in increased accumulation of reactive oxygen species (ROS) and an early leaf senescence phenotype. OsWRKY42 activates leaf senescence by repressing *OsMT1d* expression via binding to the W-box of its promoter^11^. Furthermore, OsNAP (also named PS1) acts as an important link between ABA and leaf senescence in rice. *OsNAP* transcripts are not induced by any hormone other than abscisic acid (ABA). The gain-of-function mutant *ps1* exhibits significantly premature senescence, in which expression of ABA biosynthesis genes is obviously affected. Knockdown of *OsNAP* produces an obvious delay of senescence, and most importantly, slowed down the decrease in functional photosynthetic capacity and increased the seed-setting ratio and 1,000-grain weight^12^. By contrast, expression of *OsTZF1*, a member of the CCCH-type zinc finger gene family in rice, is induced by multiple factors including drought, high-salt stress, hydrogen peroxide (H_2_O_2_), ABA, JA and salicylic acid (SA). Consistent with these findings, rice plants in which overexpression of *OsTZF1* is driven by a maize (*Zea mays*) *ubiquitin* promoter show pleiotropic phenotypes including delayed seed germination, growth retardation at the seedling stage, delayed leaf senescence, improved tolerance to high-salt and drought stresses, whereas *OsTZF1* knock-down plants exhibit much the opposite phenotypes^13^.

Thus, characterization of single SAGs has provided some insight into the complicated mechanism of leaf senescence. However, the control of gene expression during rice senescence is still under investigation. In particular, information regarding senescence-specific *cis*-elements is very limited.

Rice is a major crop that feeds about half of the human population in the world. Flag leaves are postulated to play an especially vital role in transporting nutrients into young panicles. During the grain-filling period, on one hand, photosynthesis supplies carbon components for the seeds, and on the other hand, the useful nutrition is recycled from the senescent leaves. From either perspective, the senescence of flag leaf serves as a determinant for grain yield^14^. A severe problem in facing rice breeders is premature senescence of leaves, which leads to poor grain quality and yield loss. Understanding the mechanism of flag leaf senescence and elucidating the regulatory network between the signaling pathways is essential for the improvement of grain yield.

Minghui 63 is one of the most widely used restorer lines in rice production. Previous studies showed that Minghui 63 exhibits not only good grain quality but also strong tolerance of several bacterial blight and blast diseases^15^,^16^. Here, we examined gene expression profiles in flag leaves of Minghui 63. Enrichment of transcriptional binding sites and corresponding TFs among differentially expressed genes was discovered and experimentally verified to be important for regulating leaf senescence. The data set reported herein provides an important resource for elucidating the genetic interactions between senescence signaling and regulatory responses. The identification of functional *cis-*elements may contribute to the improvement of the stay-green phenotype in plants.

## Results

### Genes showing increased or decreased transcript abundance at the onset of flag leaf senescence

**Figure 1:**
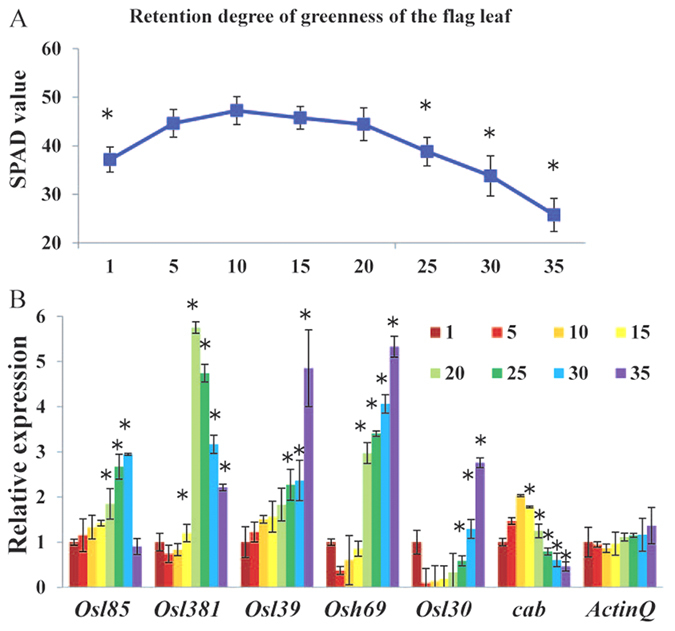
Changes of chlorophyll levels and senescence marker gene expression showing flag leaf development in Minghui 63. **(A**) Chlorophyll levels. Each bar shows the mean ± SD of five replicates. (**B**) Expression profiles of several senescence up-regulated genes and a specific senescence down-regulated gene (*cab*) reported for rice natural senescence. The numbers refer to leaf samples at 1, 5, 10, 15, 20, 25, 30 and 35 daf (days after forming 1 cm length). Each bar shows the mean ± SD of three replicates. Asterisks indicate significant differences compared to the values at 10 daf (*t*-test; P < 0.05).

First, we characterized flag leaf development to select the point of measurement for the transcriptome experiments. During leaf senescence, chlorophyll complexes are degraded, resulting in the characteristic yellowing phenomenon. Hence, we used chlorophyll levels as a marker for the phase of leaf senescence. We defined early senescence as the stage in which chlorophyll levels were 80%–90% compared to that in fully expanded green leaves, described as the S1 stage in previous reports^4^,^17^,^18^. The expression patterns of several senescence-associated genes is induced at the onset of leaf senescence, and we monitored those genes to examine the flag leaf senescence process at 1, 5, 10, 15, 20 and 25 daf (days after forming 1 cm length). *Osl85, Osl381, Osl139*, *Osh69*, and *Osl30* were used as examples of senescence-induced genes and *cab* as an example of a senescence-down regulated gene^17^. Sampled leaves were fully expanded at 5 daf, but chlorophyll levels continued to increase until 10 daf and started to drop significantly at 20 daf (Fig. 1A). The senescence marker genes were activated by 20 or 25 daf, whereas the relative transcript levels of *cab* fell steadily after 10 daf during senescence (Fig. 1B). For example, *Osl85* and *Osl381* were first up-regulated, and then declined at late senescence. *Osl39*, *Osh69* and *Osl30* showed a continued increase from 20 daf until later in senescence. Although these genes increased in expression at different time points, they showed a similar tendency of increased expression at the earlier time points of senescence. Accordingly, we retained all of these data for analysis. In field conditions, the rice plants start to head around 10 daf. Based on these results, flag leaf samples were collected at two critical stages, 5 days before heading and 14 days after heading, for data mining of early senescence-associated factors.

Affymetrix GeneChip arrays, which contain 57382 DNA oligonucleotide probe sets representing about 30000 genes per chip, were used to assay changes in the rice transcriptome in response to flag leaf senescence^19^. Overall, around 3000 genes showed up-regulation and 8000 genes showed down-regulation during senescence (http://crep.ncpgr.cn). Gene ontology (GO) analysis was used to evaluate the functional categories of genes regulated during senescence (Supplementary Fig. S1 and Table S1). TFs were extracted from the data for further analysis.

### W-box and the G-box are overrepresented in promoters of coexpressed genes

Increasing evidence indicates that genes with the similar expression patterns may contain the same regulatory elements in their promoters^5^. Accordingly, we analyzed the promoters of the up-regulated genes to identify potential TF-binding motifs important for activating flag leaf senescence. Promoters corresponding to 1 kb upstream of the predicted transcription start site of genes were screened using a new method^20^. Two known TF binding motifs, the W-box and the G-box, were found to be significantly enriched in promoters of up-regulated genes (Supplementary Table S2 and Table 1).

**Figure 2:**
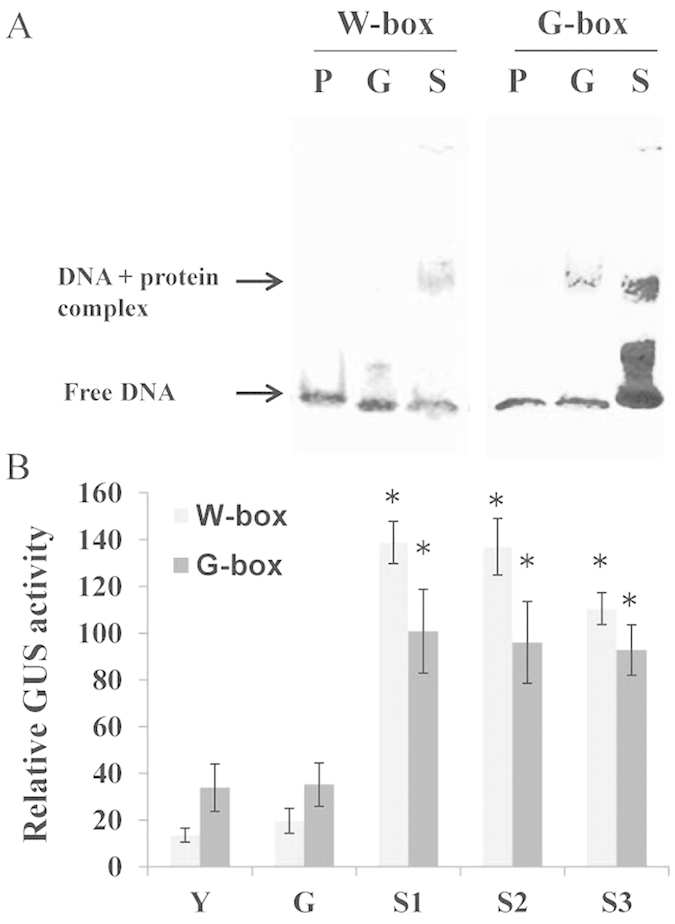
Assays of W-boxes and G-box specific activity in rice leaf senescence. (**A**) Gel retardation assay of W-box and G-box binding activity in rice nuclear protein extracts. P, free probe; G, free probe plus nuclear protein extracts from green mature leaves (3.75 mg); S, free probe plus nuclear protein extracts from senescent leaves (3.75 mg). (**B**) GUS-activity assay of W-box and G-box binding activity. Four copies of the W-box or G-box were fused to the CaMV35S minimal promoter and transformed into rice. Reporter activity was highly induced by leaf senescence. Y, young leaf; G, fully expanded green leaf; S1, senescing leaf with 80%–90% chlorophyll; S2, senescing leaf with 60%–80% chlorophyll; S3, senescing leaf with 40%–60% chlorophyll. Error bars refer to ±SE of four replicates. Asterisks indicate significant differences of indicated leaf stages to G (*t*-test; P < 0.05).

We sought to confirm the potential functional role of these motifs in leaf early senescence. Thus, we initially performed a gel shift mobility assay that involved incubating a 24-bp probe with nuclear extracts from green leaves and early senescent leaves. As shown clearly in Fig. 2A, the W-box was bound only by nuclear extracts from senescent leaves, while the G-box was bound to a greater degree by nuclear extracts of senescent leaves than by extracts of young leaves. The gel shift assays thus provided evidence that W-box and G-box elements may function to mediate the early senescence-induced expression in rice flag leaves.

To further analyze the potential activity of these elements during senescence, we generated fusions of four *cis*-element copies to the CaMV35S minimal promoter to drive expression of the *β-glucuronidase* (*GUS*) reporter gene. Four copies of the elements were used to ensure that the expression would be strong enough to be detectable by GUS staining. We carried out GUS activity assays during natural senescence of transgenic rice containing the fusions. Both elements showed little background activity in leaves, whereas GUS activity of the W-box fusion was induced 10-fold, and that of the G-box fusion 3-fold, by leaf senescence (Fig. 2). These results suggest that W-box and G-box elements play important roles in the network regulating flag leaf senescence processes *in vivo*.

### Effect of element combination in leaf senescence

**Figure 3:**
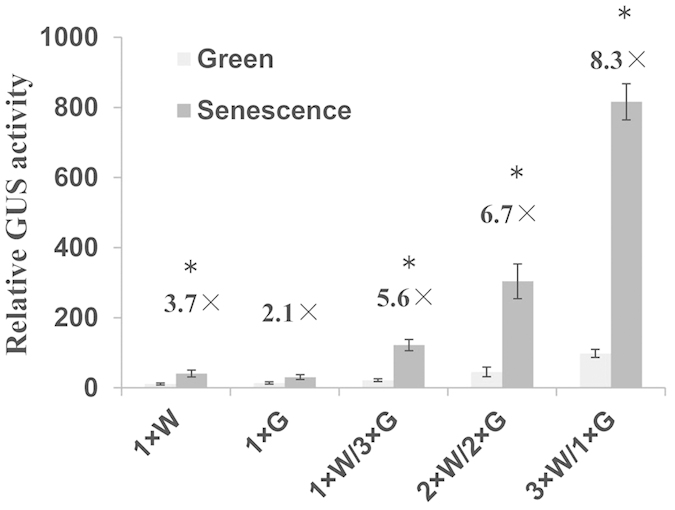
Senescence inducibility of synthetic promoters containing various numbers of W-box and G-box *cis-*elements. Light gray bars show the level of GUS activity before senescence (fully expanded green leaf). Dark gray bars represent GUS activity after senescence (senescing leaf with 60%–90% chlorophyll). The fold inducibility is shown, and error bars refer to ±SE of four replicates. Asterisks indicate significant difference before and after senescence (*t*-test; P < 0.05).

We constructed a series of synthetic promoters with GUS reporter in the backbone of pCAMBIA 1301. The promoters carried 1 × G-box, 1 × W-box, 1 × W-box with 3 × G-box, 2 × W-box with 2 × G-box or 3 × W-box with 1 × G-box. Each promoter containing a CaMV35S minimal promoter was used to test the activities of these combinations. Empty vector carrying only the CaMV35S minimum promoter fused to *GUS* was used as a control. Synthetic promoter-driven *GUS* reporter gene activity was monitored in transgenic rice during natural senescence. The plants carrying one single element gave low GUS activity during natural senescence. Interestingly, three copies of the W-box enhanced promoter expression by 6.7- and 5.6-fold compared to one and two copies of the W-box, respectively, even though background expression was apparent (Fig. 3). These results indicate that more copies of the W-box led to greater promoter activity. The highest induction of expression was obtained with 3 × W-box combined with 1 × G-box. Taken together, both W-box and G-box elements contribute to the overall expression of senescence-responsive genes and the W-box may play dominant role in response to natural senescence.

### WRKY, bZIP, bHLH and NAC families significantly overrepresented in flag leaf senescence data

In general, signaling networks involve *cis-*elements working with their cognate TFs. This raised the question of whether the TFs regulated during flag leaf senescence were consistent with the discovery of the early senescence-responsive *cis-*elements in our study. To address this question, TF families were extracted from among the differentially expressed genes based on their conserved domains.

**Figure 4:**
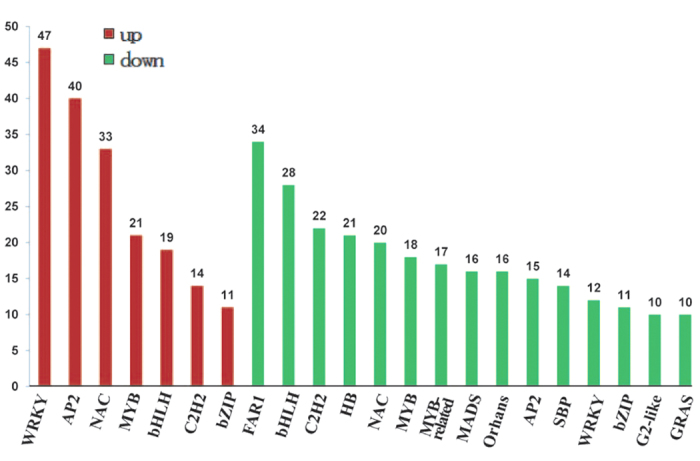
TF families significantly overrepresented among genes differentially expressed during flag leaf senescence. The number of TFs is shown above each bar. TFs up-regulated (the left 7 columns) were labeled in red and down-regulated (the right 15 columns) in green.

There were 258 up-regulated and 377 down-regulated genes encoding TFs including members of the WRKY, bZIP, NAC, MYB, and bHLH families (Supplementary Table S3 and Fig. 4). The largest group among the up-regulated TFs comprised 47 genes encoding WRKY proteins, which are generally considered as W-box binding factors. This group included OsWRKY42, which positively activates leaf senescence. Among the down-regulated TF genes were 11 belonging to the WRKY family. Thus, the WRKY family genes were significantly overrepresented in the flag leaf senescence data set, an observation that is in agreement with our data demonstrating that the W-box plays important role in senescence response regulation.

The G-box serves as a binding site for bZIP, bHLH and NAC TFs^21^. Genes for 33 NAC, 11 bZIP and 19 bHLH were enriched among the up-regulated gene pool, while those for 20 NAC, 11 bZIP and 28 bHLH were enriched in the down-regulated gene pool. These three families of proteins composed the dominant group among overrepresented TFs. This suggests that G-box functions as a critical site for regulation in leaf senescence.

### Analyses of WRKY, bZIP, bHLH and NAC in hormone responses

**Figure 5:**
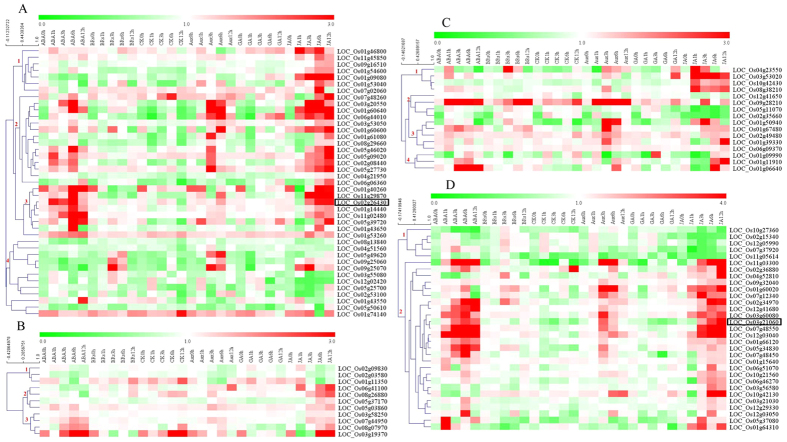
Heatmap of WRKY (**A**), bZIP (**B**), bHLH (**C**) and NAC (**D**) family members overrepresented in the set of gene up-regulated in response to ABA, CTK, BR, auxin, GA and JA. The data were obtained from the RiceXPro database. Examined genes are listed in Supplementary Table S4 and detailed information for known SAGs is shown in Supplementary Table S5. Previously known SAGs OsWRKY42 and OsNAP are encoded by LOC_Os02g26430 and LOC_Os03g21060, respectively (labeled by a black box).

Leaf senescence involves crosstalk with hormone signaling. To investigate the potential relationship between flag leaf early senescence and hormone signaling, we surveyed the responses of TF genes to abscisic acid (ABA), brassinosteroid (BR), cytokinin (CTK), auxin, JA and GA using data from RiceXPro^22^ (Supplementary Table S4 and Fig. 5). Among the TF families overrepresented in our set of differentially regulated genes, we focused on WRKY, bZIP, bHLH and NAC families. The resulting clustering showed that the four TF families were affected by various hormones to varying degrees. The TF families could be divided into several groups according to their expression patterns: 4 groups of WRKY genes, 3 groups for bZIP, 4 groups for bHLH, and 2 groups for NAC family members (Fig. 5). Group 1 of the WRKY family was highly induced by GA and JA, while WRKY groups 2 and 3 were induced by ABA, auxin and JA. Group 4 of WRKY was induced by BR and JA. Almost 90% of WRKY members were inhibited by CTK. Interestingly in the bZIP, bHLH and NAC families, members were induced by ABA, and often also induced by JA. The expression data indicate that GA and CTK are not very strong effectors for these three families. Previously reported rice SAGs were also found in this study to be related to various hormone-regulated pathways. For example, senescence positive regulator OsWRKY42 (LOC_Os02g26430) was markedly induced by ABA and JA (Han *et al.* 2014). OsNAP (Loc_Os03g21060) was induced by ABA, auxin and JA^12^,^23^. Our results demonstrate that ABA, BR and CTK-mediated signaling might converge on the same TFs.

## Discussion

Applying a novel bioinformatics algorithm, we here demonstrated on a genome-wide scale that W-box and G-box elements are significantly enriched in promoters of early senescence-inducible genes in rice flag leaf. Their related binding factors, WRKY, bZIP, bHLH and NAC family members, were overrepresented among genes differentially expressed in response to senescence.

Among the up-regulated genes during senescence were previously identified defense-activated genes in rice or homologs of those from other species, such as OsWRKY13, OsWRKY42, AtWRKY8, and WRKY5^24^,^25^,^26^ (see Supplementary Table S5 for known rice SAGs). In most cases, WRKY family TFs play a central role in the defense signaling hierarchy^27^,^28^,^29^. W-boxes are profoundly enriched in plant defense and drought resistance signaling genes^25^,^30^. There are also several examples in Arabidopsis of WRKY proteins related to senescence. High expression of the *WRKY6* TF gene leads to leaf necrosis through activating a senescence-induced receptor kinase^31^. WRKY53-overexpressing plants exhibit premature senescence, whereas *wrky53* mutants show delayed senescence^32^. It is plausible that W-box and WRKY superfamily members might function in activating leaf senescence, given that they widely participate in plant abiotic and biotic stress responses. Leaf senescence and stress responses share a common characteristic of involving cell death. It is possible that plant defense, salt stress, or drought stress activates cell death signaling.

Another important *cis*-element, the G-box, has been described to participate in light-responsive processes^33^,^34^. G-box elements are mainly present in promoters of light-responsive genes, whether positively or negatively regulated, such as *RBCS*, *CHS* and *CAB*^35^. G-box related binding factors are generally found to be members of bZIP, bHLH and NAC families^34^. Although there is much evidence demonstrating a role of phytochromes in the regulation of leaf senescence, the light-dependent signaling pathway is poorly understood. In Arabidopsis and *Petroselinum crispum* (parsley), bZIP proteins serve as regulators in UV and blue light-mediated photomorphogenesis^35^,^36^. bZIP16 and bZIP68 can form heterodimers with GBF1 (also a bZIP member) and generate light signaling outputs^37^. Interestingly, the onset of leaf senescence is delayed in the Arabidopsis *gbf1* mutant, clearly indicating a regulatory function of GBF1 in leaf senescence^38^.

Several NAC and bHLH family members have been reported to regulate senescence. In Arabidopsis, AtNAP^39^, ORE1/ONAC092^40^, and NAC016^41^ promote leaf senescence. The bHLH TFs PHYTOCHROME-INTERACTING FACTORs (PIFs) 3, 4 and 5 are early senescence genes and induce leaf senescence by activating ethylene and ABA signaling^42^,^43^. A recent study demonstrated that Arabidopsis bHLH TFs MYC2, MYC3, and MYC4 redundantly activate JA-induced leaf senescence via binding to G-box-like motifs (AACGTG and CACGTA) in the *SAG29* promoter^44^. In rice, OsNAP^12^,^23^ positively regulate leaf senescence by regulating hormone biosynthesis-related genes. In soybean, photoexcited Cryptochrome2 (CRY2a) physically interacts with CIB1 (a bHLH TF), thereby repressing the binding activity of CIB1 to the E-box (CANNTG) in the *WRKY53b* promoter, and negatively regulating leaf senescence^45^. These studies are in accordance with our results that light and flag leaf senescence signaling might converge at the G-box, and suggest that bZIP, bHLH and NAC TFs might function as upstream regulators at the transcription level.

Considering that natural senescence is a complex trait resulting from the synergy of leaf development and environmental factors, it is not surprising that light-responsive bZIP, bHLH and NAC members and the corresponding G-box binding sites were highly enriched among natural senescence-associated genes.

Natural senescence is a highly-regulated process in which the activation of signaling pathways involves the stress-related plant hormone ABA^46^,^47^. In Arabidopsis, ABA levels in leaves significantly increase at the onset of leaf senescence, with a subsequent increase to a maximum at late senescence^5^. It has been shown that ABA inhibits BR effects in response to abiotic stress^48^, whereas BR and CTK have positive interactions to mediate plant defense^49^. W-box and G-box elements both showed higher binding intensity during senescence, as well as senescence inducibility *in vivo* and *in vitro* (Fig. 3). These findings suggest that W-box and G-box elements play important roles in the regulated pathway of activating leaf senescence. Their related TFs were highly induced by ABA and JA, moderately induced by CTK and BR (Fig. 5). Moreover, CTK has been shown to activate drought resistance or delay leaf senescence in many plant species, although BR has seldom been linked to senescence^50^,^51^. In our study, up-regulated genes included a putative ABA-response gene and a gene encoding a cytokinin oxidase, but no putative BR-related genes (Supplementary Table S1). Furthermore, genes up-regulated during leaf senescence are often responsive to both JA and ABA at the same time, indicating an interesting crosstalk between these two hormone-signaling pathways (Fig. 5). In addition, our results demonstrating crosstalk between JA responses and leaf senescence are consistent with recent studies in Arabidopsis^44^ and rice^23^. Together, our findings suggest that hormone mediation of flag leaf senescence might occur through W-box and G-box elements.

A large number of plant *cis-*elements were discovered in *Arabidopsis thaliana* on the basis of gene expression patterns under multiple stresses. The copy number, location and combinatorial relationships of *cis*-elements can significantly affect the transcription of salt-responsive genes^52^. In addition, positioning an element closer to the TATA box can result in changes in promoter strength, although there is little effect on inducibility^53^. The best pathogen-inducible synthetic promoters reported consist of combinations of low copy numbers of defined *cis*-acting elements because they often combine good inducibility with low background^53^. However, the regulation of leaf senescence is quite complicated, and no data have been reported on the influence of *cis*-element combinations in the process until now. As shown in Fig. 4, the promoter with three copies of the W-box was the most induced by natural senescence compared with those with other copy numbers of W-box or G-box elements. In Arabidopsis, W-boxes and a range of *cis-*element combinations were also activated in pathogen- and wound-induced signaling, suggesting that W-box-mediated signaling is largely conserved even in different stress responses^53^.

Here, we have provided direct evidence that W-box and G-box elements can combine to mediate senescence signaling. Combination of the two *cis*-elements enhanced the expression of the *GUS* reporter gene. It was previously reported that two copies of the W-box had the greatest pathogen-specific inducibility compared to one, four, and eight copies in transient assays, even though increasing the number of copies of W-box increased the strength of the promoter^53^. These results are different from our findings, but this is perhaps not surprising because even the same individual element can behave differently in different combinations in response to stress^52^. Compared to native promoters containing *cis-*elements responsible for responsiveness to a broad range of stimuli, synthetic promoters are well defined and function more directly; moreover, the corresponding binding factors are predictable. Our findings highlight that we still have room to improve combinations of *cis*-elements to increase the efficiency of synthetic promoters for use in multiple purposes.

This report represents a first genome-wide look at *cis*-elements in flag leaf senescence including their combinatorial relationships and crosstalk with hormone signaling. W-box and G-box elements were discovered likely to be responsible for activating flag leaf senescence. Our results represent a significant step toward understanding the mechanism underlying plant flag leaf senescence and elucidate potential targets for enhancement of stay-green traits in crops.

## Methods

### Plant material and chlorophyll measurement

An indica cultivar Minghui 63 was planted in fields of the experimental farm of Huazhong Agricultural University at Wuhan, China in the 2008 rice growing season. The whole flag leaves were harvested at indicated days and frozen in liquid nitrogen for RNA or protein extraction. Chlorophyll concentration of flag leaves in Fig. 1 was measured nondestructively using a chlorophyll meter (SPAD-502; Minolta).

### Plasmid construction

The element combination carrying 4 × G-box, 4 × W-box, 1 × G-box, 1 × W-box, 1 × W-box with 3 × G-box, 2 × W-box with 2 × G-box or 3 × W-box with 1 × G-box were synthesized commercially (Sangon Biotechnology, Shanghai, China) and their sequences are as shown in Supplementary Table S6. The complement sequence for each oligo was also synthesized. All forward primers contained an *Eco*RI site at the 5′ end, and the reverse primers contained a *Bgl*II site at the 5′ end. After denaturing and annealing, double-stranded sequences were inserted between the *Spe*I and *Xba*I restriction sites upstream of the CaMV35S minimal promoter in the backbone of pCAMBIA 1301. Empty vector carrying only the CaMV35S minimum promoter fused to *GUS* was used as a control. Sequence data from this article can be found in the Michigan State University Rice Genome Annotation Project database (http://rice.plantbiology.msu.edu) under the following accession numbers: ActinQ (LOC_Os03g50885), *Osl85* (LOC_Os07g34520), *Osl381* (LOC_Os01g21320) *Osl139* (LOC_Os11g09329), *Osh69* (LOC_Os08g38710), *Osl30* (LOC_Os02g07160), and *cab* (LOC_Os06g11150).

### RNA extraction and quantitative RT-PCR assays

Total RNA was isolated from the flag leaves harvested at indicated days using TRIzol reagent (Invitrogen, Carisbad, CA, USA) according to the manufacturer’s instruction. RNA was incubated at 37 °C for 10 minutes with RNase-free DNase I (Invitrogen, USA) to eliminate genomic DNA contamination according to the manufacturer’s instructions. DNase I-treated RNA samples (3 μg) were used to generate first-strand cDNA using 200 units of SuperScript™ III reverse transcriptase (Invitrogen, USA) following the protocol described above. To determine the expression of candidate genes, Quantitative RT-PCR (qRT-PCR) was performed as described previously^29^ by using 1μl of the diluted cDNA as a template combined with control primer and the gene-specific primer pairs in a total volume of 25 μl for the PCR reactions. The actin transcripts served as internal controls. Gene-specific qRT-PCR primers were designed based on the cDNA sequences and were synthesized commercially (Sangon Biotechniques, shanghai, China).The sequences of the primers are listed in Supplementary Table S6. Values are means ± SD of three biological repeats. *T*-test was used for statistical analysis.

### Gel shift assays

Young or senescent leaves were harvested and nuclear extracts were isolated using the method reported before^54^. Two DNA probes were synthesized commercially (Sangon Biotechnology, Shanghai, China) using the following sequences: 4 × W-box, 5′-TTGACTTTGACCTTGACTTTGACC-3′; 4 × G-box, 5′-CACGTGCACGTGCACGTGCACGTG-3′. The complement sequence for each oligo was also synthesized. After denaturing and annealing, double-stranded probes were used for labeling. Gel mobility shifts were performed as described previously^54^.

### Data analysis

The methodology and data used in this paper were originally from National Center of Plant Gene Research (Wuhan) (http://crep.ncpgr.cn). The array experiments were carried out using the Affymetrix rice whole genome chip containing 57382 probes representing approximately genes. The data was released in Wang *et al.*^15^ using Affymetrix rice whole genome chip to study gene expression patterns for leaf senescence. The list of differentially expressed genes is shown in Supplementary Table S1. Promoters corresponding to 1 kb upstream of the predicted transcription start site of genes were screened using the method reported in Ma *et al.*^20^. We analyzed the chances of the observed distribution of the elements in our promoters occurring by chance. The *cis*-elements with P-value <0.01 are defined to be enriched. The rice upstream sequences were downloaded from the Rice Annotation Project Database (http://rapdb.dna.affrc.go.jp/). The IRGSP/RAP build 5 dataset was used to extract the 1000 base pair sequences upstream of 5′ UTR from those genes with 5′ UTR longer than 50 bp. Enriched Gene ontology (GO) terms were identified using WEGO^55^.

### Plant transformation and GUS activity

The various constructs described above were transferred into Agrobacterium tumefaciens strain EHA105 by electroporation. The resulting strains were then transferred into Zhonghua 11 (O. sativa sp. japonica) by Agrobacterium-mediated transformation^18^. The positive transformants were selected by PCR using the hygromycin phosphotransferase gene-specific primers hpt-F (5′-ATTTGTGTACGCCCGACAGT-3′) and hpt-R (5′-GGATATGTCCTGCGGGTAAA-3′). GUS activity was measured before and after senescence, generating a relative expression value for each treatment. Each GUS activity was measured using three samples, with each sample containing ten plants. Histochemical staining and quantitative analyses of GUS activity were conducted as described previously^56^.

## Additional Information

**How to cite this article**: Liu, L. *et al.* W-box and G-box elements play important roles in early senescence of rice flag leaf. *Sci. Rep.* **6**, 20881; doi: 10.1038/srep20881 (2016).

## Supplementary Material

Supplementary Information

Supplementary Dataset 1

## Figures and Tables

**Table 1:** Distribution and frequency of W-box and G-box in promoters of genes up-regulated in rice flag leaf during senescence.

Motif	Instance in genome	Mean position	Instance in cluster	Mean position	P-Value
TTGACY	32162	−500	1139	−455	4.34E-09
TTGAC	60612	−505	1985	−471	1.27E-05
CACGTG	47436	−430	1544	−357	0.000255
TTTGACY	14857	−505	509	−468	0.000917

The first, second and fourth rows show the analysis for W-box, and the third row shows the analysis for G-box.
