# Supplementary material for: W-box and G-box elements play important roles in early senescence of rice flag leaf
Source: Sci Rep. 2016 Feb 11;6:20881. doi: 10.1038/srep20881 (PMC4749992; doi:10.1038/srep20881)
Supplement: Supplementary Information [file srep20881-s1.pdf]

W-box and G-box elements play important roles in early senescence of rice flag leaf

Li Liu[1,2*], Wei Xu[1,2], Xuesong Hu[3], Haoju Liu[4,5] & Yongjun Lin[5*]

[1]Key Laboratory for Economic Plants and Biotechnology, Kunming Institute of Botany, Chinese Academy of Sciences, Kunming, 650201, China

[2]Yunnan Key Laboratory for Wild Plant Resources, Kunming, 650201, China

[3]College of Life Sciences, Peking-Tsinghua Center for Life Sciences, Peking University, Beijing, 100871, China

[4]Department of Bioscience and Bioengineering, Jiangxi Agricultural University, Nanchang, 330045, China

[5]National Key Laboratory of Crop Genetic Improvement and National Centre of Plant Gene Research, Huazhong Agricultural University, Wuhan, 430070, China

To whom correspondence should be addressed. Email: liulia@mail.kib.ac.cn or yongjunlin@mail.hzau.edu.cn

Supplementary Figure S1. Functional classification of leaf senescence-associated genes in rice flag leaf. The distribution of GO terms in each category is shown for genes up-regulated (red) and down-regulated (gray) during senescence of flag leaves. Enriched GO terms were identified using WEGO[55].

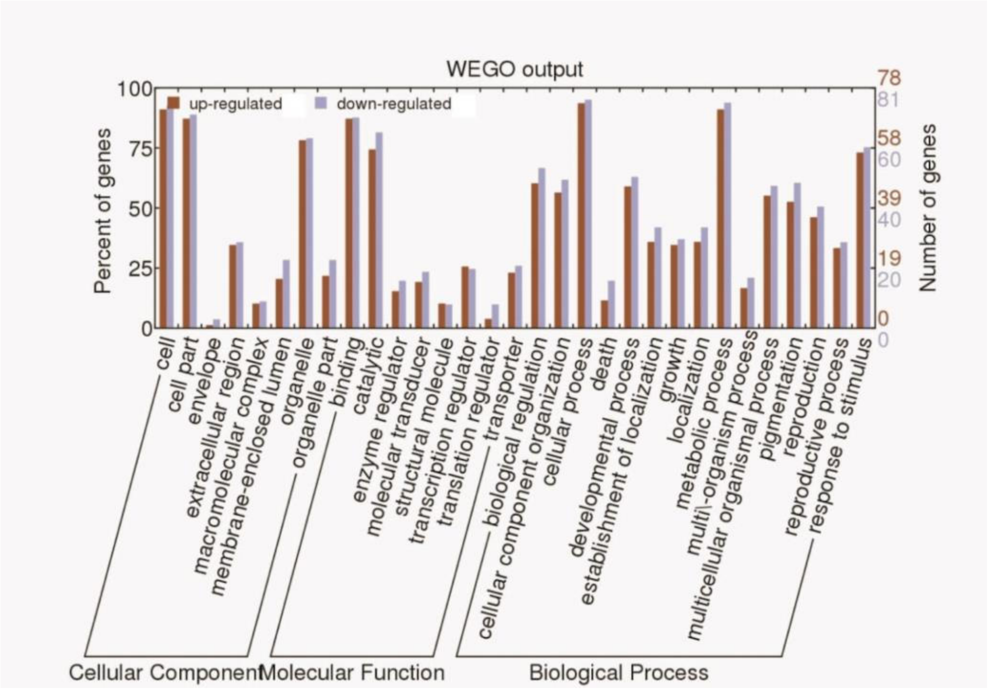

Supplementary Table S5. Expression pattern of known rice SAGs in flag leaf microarray data. Asterisks indicate significant differences before to after senescence (*t*-test; P < 0.05).

Name	Gene ID	Treatment		Reference
		5 days	14 days	
OsSGR*	LOC_Os09g36200	151 ± 2	1043 ± 66	1
OsNYC4*	LOC_Os07g37250	10434 ± 883	4652 ± 65	2
OsRab7B3*	LOC_Os05g44050	10465 ± 1036	12908 ± 454	3
OsTZF1*	LOC_Os05g10670	3459 ± 95	866 ± 473	4
OscZOGT1	LOC_Os04g46980	21 ± 0	57 ± 20	5
OsNYC3*	NM_001064128	3299 ± 189	5097 ± 959	6
OsDOS	NM_001048811	17 ± 12	15 ± 0	7
OsDWARF3*	AK069429	242 ± 20	61 ± 17	8
OsTDC1	AK069031	20 ± 15	82 ± 17	9
OsSPL28	LOC_Os01g50770	2859 ± 296	2747 ± 267	10
OsETR2*	AF420319	776 ± 110	580 ± 10	11
OsAkαGal*	AF251068	369 ± 171	5044 ± 1249	12
OsGS1;3	AB180689	33 ± 22	68 ± 11	13
OsPHYB	AB109892	5513 ± 623	2957 ± 186	14
OsGS1;1	AB037595	6045 ± 535	9738 ± 315	15
OsNOE1	LOC_Os03g03910	27930 ± 3278	24420 ± 1667	16
OsNAP*	LOC_Os03g21060	309 ± 69	4069 ± 1087	17
OsWRKY42*	LOC_Os02g26430	22 ± 11	2160 ± 714	18
OsNYC1*	LOC_Os01g12710	10101 ± 400	5845 ± 252	19
OsSWEET5	LOC_Os05g51090	41 ± 19	56 ± 8	20
OsCOI1b	LOC_Os05g37690	6040 ± 81	5994 ± 761	21

References

1. Park, S.Y., *et al.* The senescence-induced staygreen protein regulates chlorophyll degradation. *Plant Cell* **19**, 1649-1664 (2007).
2. Yamatani, H., *et al.* NYC4, the rice ortholog of Arabidopsis THF1, is involved in the degradation of chlorophyll protein complexes during leaf senescence. *Plant J* **74**, 652-662 (2013).
3. Sugunya, P., *et al.* Overexpression of OsRab7B3, a Small GTP-Binding Protein Gene, Enhances Leaf Senescence in Transgenic Rice. *Biosci Biotech Biochem* **76**, 1296-1302 (2012).
4. Jan, A., *et al.* OsTZF1, a CCCH-tandem zinc finger protein, confers delayed senescence and stress tolerance in rice by regulating stress-related genes. *Plant Physiol* **161**, 1202-1216 (2013).
5. Kudo, T., *et al.* Cytokinin activity of cis-zatin and phenotypic alterations induced by overexpression of putative cis-zeatin-O-glucosyltransferase in rice. *Plant Physiol* **160**, 319-331 (2012).
6. Morita, R., *et al.* Defect in non-yellow coloring 3, an alpha/beta hydrolase-fold family protein, causes a stay-green phenotype during leaf senescence in rice. *Plant J* **59**, 940-952 (2009).
7. Kong, Z.S., *et al.* A novel nuclear-localized CCCH-type zinc finger protein, OsDOS, is involved in delaying leaf senescence in rice. *Plant Physiol* **141**, 1376-1388 (2006).
8. Yan, H., *et al.* Rice tillering dwarf mutant dwarf3 has increased leaf longevity during darkness-induced senescence or hydrogen peroxide-induced cell death. *Genes Genet Syst* **82**, 361-366 (2007).
9. Kang, K., *et al.* Senescence-induced serotonin biosynthesis and its role in delaying senescence in rice leaves. *Plant Physiol* **150**, 1380-1393 (2009).
10. Qiao, Y., *et al.* SPL28 encodes a clathrin-associated adaptor protein complex 1, medium subunit μ1 (AP1M1) and is responsible for spotted leaf and early senescence in rice (*Oryza sativa*). *New Phytol* **185**, 258-274 (2010).
11. Wuriyanghan, H., *et al.* The ethylene receptor ETR2 delays floral transition and affects starch accumulation in rice. *Plant Cell* **21**, 1473-1494 (2009).
12. Lee, R.H., *et al.* Alkaline alpha-galactosidase degrades thylakoid membranes in the chloroplast during leaf senescence in rice. *New Phytol* **184**, 596-606 (2009).
13. Cai, H.M., *et al.* Overexpressed glutamine synthetase gene modifies nitrogen metabolism and abiotic stress responses in rice. *Plant Cell Rep* **28**, 527-537 (2009).
14. Piao, W., *et al.* Rice Phytochrome B (OsPhyB) Negatively Regulates Dark-and Starvation-Induced Leaf Senescence. *Plants* **4**, 644-663 (2015)
15. Tabuchi, M., *et al.* Severe reduction in growth rate and grain filling of rice mutants lacking OsGS1;1, a cytosolic glutamine synthetase1;1. *Plant J* **42**, 641-651 (2005).

16. Lin, A.H., *et al*. Nitric Oxide and Protein S-Nitrosylation Are Integral to Hydrogen Peroxide-Induced Leaf Cell Death in Rice. *Plant Physiol* **158**, 451-464 (2012).
17. Liang, C.Z., *et al*. OsNAP connects abscisic acid and leaf senescence by fine-tuning abscisic acid biosynthesis and directly targeting senescence-associated genes in rice. *Pro Nat Acad Sci USA* **111**, 10013-10018 (2014).
18. Han, M., *et al*. OsWRKY42 Represses OsMT1d and Induces Reactive Oxygen Species and Leaf Senescence in Rice. *Mol Cells* **37**, 532-539 (2014).
19. Kusaba, M., *et al*. Rice NON-YELLOW COLORING1 Is Involved in Light-Harvesting Complex II and Grana Degradation during Leaf Senescence. *Plant Cell* **19**, 1362-1375 (2007).
20. Zhou, Y., *et al*. Overexpression of OsSWEET5 in Rice Causes Growth Retardation and Precocious Senescence. *Plos One* **9,** (2014).
21. Lee, S.H., *et al*. Mutation of Oryza sativa CORONATINE INSENSITIVE 1b (OsCOI1b) delays leaf senescence. *J Integr Plant Biol* **57**, 562-576 (2015).
